# Herpesvirus genome integration in whole‐genome sequences of dementia and control cohorts

**DOI:** 10.1002/alz.71047

**Published:** 2026-03-19

**Authors:** Stacey L. Piotrowski, Mary Alice Allnutt, Kory Johnson, Toshiko Tanaka, Luigi Ferrucci, Huw Morris, John Hardy, PROSPECT Consortium, Mina Ryten, United Kingdom Brain Expression Consortium (UKBEC), Giancarlo Logroscino, Juan Troncoso, Thomas G. Beach, Geidy E. Serrano, Carlos Cruchaga, Dennis W. Dickson, Owen A. Ross, Adriano Chiò, International LBD Genomics Consortium, International ALS Genomics Consortium, Henry Houlden, The American Genome Center, Clifton L. Dalgard, Jinhui Ding, J. Raphael Gibbs, Bryan J. Traynor, Sonja W. Scholz, Steven Jacobson

**Affiliations:** ^1^ Viral Immunology Section National Institute of Neurological Disorders and Stroke National Institutes of Health Bethesda Maryland USA; ^2^ Bioinformatics Core National Institute of Neurological Disorders and Stroke National Institute of Health Bethesda Maryland USA; ^3^ Translational Gerontology Branch National Institute on Aging National Institutes of Health Baltimore Maryland USA; ^4^ Department of Clinical and Movement Neurosciences UCL Queen Square Institute of Neurology Royal Free Hospital London UK; ^5^ UCL Movement Disorders Centre University College London Royal Free Hospital London UK; ^6^ UK Dementia Research Institute and Department of Neurodegenerative Disease and Reta Lila Weston Institute UCL Queen Square Institute of Neurology and UCL Movement Disorders Centre University College London Institute of Neurology, Queen Square London UK; ^7^ Institute for Advanced Study The Hong Kong University of Science and Technology Hong Kong SAR, Lo Ka Chung Building Lee Shau Kee Campus The Hong Kong University of Science and Technology Clear Water Bay Kowloon Hong Kong; ^8^ NIHR Great Ormond Street Hospital Biomedical Research Centre University College London London UK; ^9^ Department of Genetics and Genomic Medicine Great Ormond Street Institute of Child Health University College London London UK; ^10^ Department of Translational Biomedicine and Neuroscience University of Bari Aldo Moro Bari BA Italy; ^11^ Center for Neurodegenerative Diseases and the Aging Brain University of Bari Aldo Moro at Pia Fondazione “Card. G. Panico” Tricase Italy; ^12^ Department of Neurology Johns Hopkins University Medical Center Baltimore Maryland USA; ^13^ Department of Pathology Johns Hopkins University School of Medicine Baltimore Maryland USA; ^14^ Civin Laboratory for Neuropathology Banner Sun Health Research Institute Sun City Arizona USA; ^15^ Department of Psychiatry Washington University in St Louis St Louis Missouri USA; ^16^ NeuroGenomics and Informatics Center Washington University School of Medicine St. Louis Missouri USA; ^17^ Department of Neuroscience Mayo Clinic Florida Jacksonville Florida USA; ^18^ Department of Clinical Genomics Mayo Clinic Jacksonville Florida USA; ^19^ “Rita Levi Montalcini” Department of Neuroscience University of Turin Torino (TO) Italy; ^20^ Institute of Cognitive Sciences and Technologies C.N.R. Roma (RM) Italy; ^21^ Azienda Ospedaliero Universitaria Città della Salute e della Scienza Torino (TO) Italy; ^22^ Department of Neuromuscular Diseases University College London Queen Square Institute of Neurology Queen Square House London UK; ^23^ The National Hospital for Neurology and Neurosurgery, Queen Square London UK; ^24^ Department of Anatomy Physiology and Genetics Uniformed Services University of the Health Sciences Bethesda Maryland USA; ^25^ Computational Biology Group Laboratory of Neurogenetics National Institute on Aging Bethesda Maryland USA; ^26^ Laboratory of Neurogenetics National Institute on Aging National Institutes of Health Bethesda Maryland USA; ^27^ Neurodegenerative Diseases Research Section National Institute of Neurological Disorders and Stroke Bethesda Maryland USA

**Keywords:** dementia, herpesviruses, human herpesvirus 6, neurodegenerative disease

## Abstract

**INTRODUCTION:**

The infectious hypothesis suggests that microbes like herpesviruses may play a role in the pathogenesis of Alzheimer's disease (AD) and other related dementias through methods that may include viral genome integration. The occurrence of herpesvirus genome integration in dementia patients has not been thoroughly characterized.

**METHODS:**

Over 7500 total whole‐genome sequences from control, frontotemporal dementia/amyotrophic lateral sclerosis spectrum, Lewy body dementia (LBD), multiple system atrophy (MSA), and AD cohorts were screened for the integration of pathogen genomes using the PathSeq computational tool.

**RESULTS:**

Low PathSeq scores for human herpesvirus 6 (HHV‐6) were consistent with the suspected integration of viral genome segments. The LBD and MSA cohorts had a significantly higher prevalence of this partial HHV‐6 genome integration.

**DISCUSSION:**

This higher prevalence in both synucleinopathies was not noted in other herpesviruses, suggesting that the integration of HHV‐6 may play a role in a subset of these patients.

**Highlights:**

- Over 7500 whole‐genome sequences from controls and dementia patients were analyzed.
- Sequences consistent with integrated herpesviruses were identified using PathSeq.
- Prevalence of partial HHV‐6 integration was higher in synucleinopathies.
- Herpesviruses genome integration may play a role in subsets of dementia patients.

## 1 BACKGROUND

Infectious agents, like viruses, have long been suspected of contributing to neurodegenerative diseases, such as Alzheimer's disease (AD) and related dementias.^1^, ^2^ In many of these disorders, viruses are thought to potentially contribute to the pathogenesis through a variety of mechanisms, including neuroinflammation, reactivation from viral latency, integration of genome sequences, and the direct seeding of proteins such as amyloid beta (Aβ).^3^, ^4^, ^5^, ^6^, ^7^, ^8^, ^9^, ^10^, ^11^ In particular, the interest in a possible association between neurotropic herpesviruses and progressive neurologic diseases that result in dementia continues to grow.^1^, ^2^, ^12^, ^13^, ^14^, ^15^, ^16^, ^17^, ^18^, ^19^

Some herpesviruses have been shown to have the ability to integrate into host genomes either incidentally or as part of their life cycle.^8^ Unlike most herpesviruses, which maintain episomes, human herpesvirus 6 (HHV‐6) achieves latency through the integration of its viral genome into the host cell genome.^20^, ^21^, ^22^ HHV‐6 has been shown to integrate into the telomeres of human host cells through homologous recombination via direct repeat (DR) regions on the ends of the viral genome.^23^, ^24^ This integration can occur in small numbers of somatic cells in infected individuals, such as T cells or glial cells.^21^, ^22^, ^25^ When integration of the full‐length viral genome occurs in germ cells, it can be vertically transmitted from parent to offspring as inherited chromosomally integrated HHV‐6 (iciHHV‐6), where the complete viral genome is integrated into a chromosome of every nucleated cell in the body.^22^, ^26^, ^27^, ^28^ In such cases, the affected individual has a high viral load of approximately one copy per cell.^22^, ^26^, ^27^, ^28^ Moreover, iciHHV‐6 is present in as high as 1% of the general population, with studies suggesting it may be a risk factor for certain pathologies, including angina pectoris, spontaneous abortion, and preeclampsia.^26^, ^29^, ^30^, ^31^ Reactivation of iciHHV‐6 has been reported *in vitro;* however, the significance of this reactivation in human disease is unknown.^20^, ^30^ Recently, the integration of only portions of the virus, such as the DR regions, has also been documented.^32^ While much less common, incidental integration of other herpesviruses has also been previously reported, including Epstein‐Barr virus (EBV), herpes simplex virus 1 (HSV‐1), and varicella zoster virus (VZV).^8^, ^33^

The role of herpesviruses in neurodegenerative diseases that culminate in dementia remains unclear, with some research suggesting pathogens like HSV‐1 and HHV‐6 may play a part in AD, while others fail to find evidence of an association between disease and herpesvirus.^5^, ^12^, ^16^, ^18^, ^34^, ^35^, ^36^, ^37^ Despite the recent interest in the relationship between herpesviruses and these diseases, the possibility that herpesvirus genome integration, including both full‐length genomes like iciHHV‐6 and integration of portions of viral genomes, may contribute to disease pathogenesis has not been thoroughly investigated. To further evaluate the possible role of herpesvirus genome integration in neurodegenerative diseases associated with dementia, we accessed over 7500 total whole‐genome sequences (WGSs) for this study, including neurologically healthy control individuals and patients diagnosed with frontotemporal dementia/amyotrophic lateral sclerosis (FTD/ALS) spectrum, Lewy body dementia (LBD), multiple system atrophy (MSA), and AD. These WGSs were analyzed using the Broad Institute PathSeq tool, which removes host reads and compares the remaining reads to over 25,000 reference microbial sequences, including neurologically relevant herpesviruses such as HHV‐6.^34^, ^38^, ^39^ Droplet digital polymerase chain reaction (ddPCR), a highly sensitive PCR assay, was utilized to confirm iciHHV‐6 in a subset of samples with high PathSeq scores for HHV‐6.^40^ While iciHHV‐6 was found in cohorts at generally expected rates, the prevalence of individuals with low positive PathSeq scores that may be indicative of the integration of HHV‐6 genome segments or partial HHV‐6 integration was significantly higher in LBD and MSA cohorts. Additionally, this higher prevalence in synucleinopathies was specific for HHV‐6 and not noted in the other herpesviruses of interest in this study. These observations suggest that the genome integration of herpesviruses, particularly HHV‐6, may play a role in a subset of LBD and MSA patients.

## 2 METHODS

### 2.1 Subjects and WGSs

The appropriate Institutional Review Boards of participating institutions approved the study (National Institutes of Health, National Institute on Aging, study number: 03‐AG‐N329), and informed consent was obtained from all subjects or their surrogate decision‐makers, according to the Declaration of Helsinki.

Participants were recruited across European and North American centers. Cohorts were composed of males and females 18 years of age or older. Individuals with other concurrent neurological conditions, such as stroke or severe microvascular disease, that could mimic LBD, FTD/ALS, MSA, or AD or affect the interpretation of the study were excluded. Patients with non‐Caucasian ethnic backgrounds were also excluded. Affected patients were diagnosed according to their disease‐specific consensus criteria (Table 1). The control cohort participants were selected based on a lack of evidence of cognitive decline in their clinical history and absence of neurological deficits on neurological examination. Pathologically confirmed control subjects had no evidence of significant neurodegenerative disease on histopathological examination.

**TABLE 1 alz71047-tbl-0001:** Whole‐genome sequences (WGS) from control and dementia cohorts were analyzed in this study.

Cohort	Number of Whole Genome Sequences (WGS)	Source of WGS	Method of diagnosis
Neurologically normal controls^a^ *n* = 2202	1679 (76%) 523 (24%)	Blood Cerebellum	Clinical Pathological
Frontotemporal dementia/amyotrophic lateral sclerosis spectrum (FTD/ALS) *n* = 2372	2,184 (92%) 188 (8%)	Blood Cerebellum	Clinical^b^ Pathological^c^
Lewy body dementia (LBD) *n* = 2340	4 (0.2%) 2336 (99.8%)	Blood Cerebellum	Clinical^d^ Pathological^e^
Multiple system atrophy (MSA) n = 506	506 (100%)	Cerebellum	Pathological^f^
Alzheimer's disease (AD) *n* = 127	36 (28%) 91 (72%)	Blood Cerebellum	Clinical^g^ Pathological^h^

^a^ Neurologically healthy at the time of sample collection.

^b^ Clinically diagnosed with frontotemporal dementia spectrum disorders.^41^, ^42^, ^43^ ^.^

^c^ Pathologically confirmed FTD‐TAR DNA‐binding protein (TDP) and/or FTD‐tau.

^d^ Clinically diagnosed probable dementia with Lewy bodies or clinically diagnosed probable Parkinson's disease dementia as defined by consensus criteria.^45^, ^46^ ^.^

^e^ Pathological diagnosis of intermediate or high likelihood for dementia with Lewy bodies as defined by consensus criteria.^45^ ^.^

^f^ Pathologically definite cases of MSA using consensus criteria.^47^ ^.^

^g^ Clinically diagnosed cases of Alzheimer's disease using published guidelines.^49^ ^.^

^h^ Pathologically confirmed cases of Alzheimer's disease using published guidelines^48^ ^.^

A total of 7547 WGS were analyzed in this study (Table 1). WGS was generated using a uniform library preparation and sequencing pipeline. Briefly, PCR‐free, non‐indexed libraries from genomic DNA samples were constructed using the Illumina TruSeq DNA library kit (Illumina Corp., San Diego, CA, USA). Genome sequencing was performed on an Illumina HiSeq X10 sequencing platform using 150 base‐pair, paired‐end cycles. The mean sequencing coverage of the samples was 35×, and 2202 WGSs from neurologically healthy controls were sourced from the blood (*n* = 1679; 76%) or cerebellum (*n* = 523; 24%). In addition, 2372 FTD/ALS WGSs were mostly derived from the blood of patients that were clinically diagnosed with FTD/ALS spectrum (*n* = 2184; 92%),^41^, ^42^, ^43^, ^44^ with a small number of WGSs from the cerebellum of patients with pathologically confirmed FTD‐TAR DNA‐binding protein (TDP) and/or FTD‐tau (*n* = 188; 8%). Almost all 2340 LBD WGSs were sourced from the cerebellum of patients with pathologically diagnosed disease defined by consensus criteria (*n* = 2336; 99.8%), with few WGSs from the blood of individuals clinically diagnosed with probable dementia with Lewy bodies or Parkinson's disease dementia (*n* = 4; 0.2%).^45^, ^46^ All (100%) 506 MSA WGSs were extracted from the cerebellum of pathologically definite cases using the Gilman consensus criteria.^47^ One hundred twenty‐seven WGSs from AD patients were sourced from the cerebellum of pathologically confirmed cases (*n* = 91; 72%)^48^ or the blood of clinically diagnosed patients (*n* = 36; 28%).^49^

**RESEARCH IN CONTEXT**

1. **Systematic review**: The authors reviewed the literature using traditional sources (e.g., PubMed and Google Scholar). While the role of herpesviruses in neurodegenerative diseases such as AD has long been investigated, the unique ability of some herpesviruses to integrate into host genomes is often not considered. The prevalence of herpesvirus genome integration has not been thoroughly characterized in dementia populations.
2. **Interpretation**: Using the PathSeq tool, WGSs from dementia and control cohorts were analyzed for possible integrated herpesvirus genomes. Regions consistent with integrated segments of HHV‐6 were identified. The LBD and MSA cohorts had a significantly higher prevalence of this suspected partial HHV‐6 integration compared to the control cohort, suggesting that it may play a role in a subset of synucleinopathy patients.
3. **Future directions**: Analysis of WGSs from additional neurodegenerative disease cohorts, including a larger number of AD patients, will further characterize the prevalence of integrated herpesvirus genomes in these disease populations. Future studies may confirm and identify the sites of suspected HHV‐6 partial integration in the human genome and help establish how it occurs and the role it may be playing in disease pathogenesis.

Raw sequence data in FASTQ format were transferred to Google Cloud Storage and processed following the pipeline standard developed by the Centers[Table alz71047-tbl-0001] for Common Disease Genomics (CCDG: https://www.genome.gov/Funded‐Programs‐Projects/NHGRI‐Genome‐Sequencing‐Program/Centers‐for‐Common‐Disease‐Genomics). The genomes were aligned to the GRCH38DH reference genome using the Broad Institute's implementation of the functional equivalence standardized pipeline, which incorporates the Genome Analysis Toolkit (GATK) (2016) Best Practices.^50^

For sample‐level quality control, we excluded genomes for the following reasons: high contamination rate (>5% based on the VerifyBamID freemix metric), excessive heterozygosity rate (exceeding ± 0.15 F‐statistic), low call rate (≤95%), discordance between reported sex and genotypic sex, or duplicate samples (determined by the pi‐hat statistic), as described elsewhere.^44^, ^51^

### 2.2 Screening for herpesvirus genome integration in WGSs using PathSeq

The PathSeq tool was developed by the Broad Institute and is publicly available as a tool suite in the GATK.^38^, ^39^ WGSs were screened for a total of 25,917 microbes via PathSeq, including neurologically relevant herpesviruses: human alphaherpesvirus 1 (herpes simplex virus type 1, HSV‐1), human alphaherpesvirus 2 (herpes simplex virus type 2, HSV‐2), human alphaherpesvirus 3 (VZV), human betaherpesvirus 5 (human cytomegalovirus, HCMV), human betaherpesvirus 6A (HHV‐6A), human betaherpesvirus 6B (HHV‐6B), human betaherpesvirus 7 (HHV‐7), and human gammaherpesvirus 4 (EBV).

PathSeq utilizes computational subtraction to identify non‐human nucleic acids that may be consistent with microbial sequences.^38^, ^39^ PathSeq scores are based on the number of reads in a WGS that align with the taxon reference sequences, indirectly indicating the abundance of that microbe in the analyzed sample and correlating pathogen load with a score that is based on DNA read counts.^34^, ^38^ For example, reads with a single valid alignment result in a score of one for the corresponding species.^34^, ^39^ In this study, WGSs with a PathSeq score greater than 0 for a given pathogen were considered positive for that agent. Unlike other herpesviruses, positive PathSeq scores for HHV‐6 were stratified based on the magnitude of the score: Over 8000 was considered a high score, and under 8000 was considered a low score.

### 2.3 Confirming iciHHV‐6 using ddPCR

We had access to the DNA extracted from the blood or cerebellum for a subset of control (*n* = 17), LBD (*n* = 13), and MSA subjects (*n* = 1), which were analyzed to confirm the presence of iciHHV‐6 using a previously described ddPCR assay.^34^, ^40^ Briefly, primers to amplify the U57 major capsid protein of HHV‐6A and HHV‐6B were utilized with primers for ribonuclease P protein subunit P30 (RPP30), which was used as a reference housekeeping gene since diploid cells contain two copies. Probes were fluorescently labeled, with HHV‐6A and HHV‐6B probes fluorescein amidite (FAM)‐MGBNFQ‐labeled, while RPP30 probes were VIC‐MGBNFQ‐labeled. For each DNA sample, primers and probes for HHV‐6A or HHV‐6B were duplexed with RPP30, with the final concentrations of 900 nM per primer and 250 nM per probe.

Each sample was analyzed using duplicate wells. Fluorescence data for each well were analyzed using QuantaSoft software, version 1.7.4.0917 (Bio‐Rad, Hercules, CA, USA). Droplet positivity was determined by fluorescence intensity, with droplets above a manually determined minimum amplitude threshold considered positive. For each sample, target copies per microliter were calculated by averaging replicate wells, and cellular DNA input was calculated by halving the number of RPP30 copies to account for two copies of RPP30 per diploid cell. Results are reported as copies of virus per cell, with approximately one copy of HHV‐6A or HHV‐6B per cell, consistent with iciHHV‐6.^34^, ^40^, ^52^

### 2.4 Characterizing viral genome segments present in WGSs via Integrative Genomics Viewer

A subset of WGSs was analyzed using Integrative Genomics Viewer (IGV) version 2.8.12, developed by the Broad Institute, in order to visualize the portions of the HHV‐6 genome that were present in the WGS.^53^ HHV‐6A reference genome NC_001664.4 and HHV‐6B reference genome NC_000898.1 were used. Individual alignment. bam files were sorted and indexed using the sort command followed by the index command in igvtools within IGV. Sorted.bam files for WGSs were uploaded into IGV and compared to HHV‐6 reference genomes, providing a coverage track to view the depth of coverage. The coverage track calculates coverage for an alignment file and displays the number of reads at each locus as a bar graph.^53^ These bars are gray if they match the reference genome; colors are used to highlight mismatches to the reference genome.^53^

### 2.5 Statistical analyses

The prevalence of integration of each neurologically relevant herpesvirus was determined in each cohort by calculating the percentage of individuals with positive PathSeq scores for the given herpesvirus or, in the case of HHV‐6, the percentage of individuals with high HHV‐6 PathSeq scores and then low HHV‐6 PathSeq scores. Contingency tables were constructed for positive and negative PathSeq scores for each herpesvirus of interest, including separate high and low score tables for HHV‐6 for each cohort. Fisher's exact tests were performed on these contingency tables to determine if there was any relationship between PathSeq scores for each herpesvirus and dementia classification and if there were any significant differences in prevalence between the control and disease cohorts. Unpaired two‐tailed *t* tests were also performed to determine any significant differences in the means of the positive PathSeq scores between control and disease cohorts. These statistical analyses were performed using GraphPad Prism version 9.3.1 (GraphPad Software, San Diego, CA, USA).

To determine and control for the effects of covariates such as age, sex, WGS tissue source/diagnosis (blood source = clinical diagnosis, cerebellum source = pathological diagnosis), and country of sample collection, ANCOVA analysis was performed. The main ANCOVA model used for Akaike information criterion (AIC)‐step optimization for each herpesvirus of interest was PathSeq scores ∼ Cohort Classification coded by HHV‐6 status (no HHV‐6 detected by PathSeq, low HHV‐6 PathSeq score, or high HHV‐6 PathSeq score) + Sex + Age + Source + Country. After optimization, post hoc testing was performed using the optimized model and the adjusted scores.

## 3 RESULTS

### 3.1 PathSeq identified integrated herpesvirus genomes in dementia and control cohorts

In this study, a total of 7547 chromosome‐associated WGS from control, FTD/ALS, LBD, MSA, and AD cohorts were analyzed for evidence of possible microbial genome integration via the PathSeq pipeline (Table 1). PathSeq subtracts input reads that align to human reference sequences.^38^, ^39^ It then aligns the remaining reads to a large microbial reference sequence library (available for download at https://data.ninds.nih.gov/). In particular, we were interested in herpesvirus‐associated PathSeq scores, as herpesviruses have been reported to play a role in many chronic, progressive neurologic diseases.^1^, ^2^, ^37^ The resulting PathSeq scores for the WGSs are based on the number of reads that align with the given herpesvirus reference sequences (Data S1).^38^, ^39^ While the vast majority (5612/7547, 74%) of these WGSs had PathSeq scores of 0, suggestive of no viral genome integration, for all herpesviruses of interest, any PathSeq scores greater than 0 were considered positive for that respective pathogen, with potential integration of that viral genome present in the human WGS.

ANCOVA analysis was performed to determine any potential effects of covariates such as age, sex, WGS source, and location of sample collection on mean PathSeq scores (Data S2). Due to the three classifications of HHV‐6 PathSeq scores noted in this study (“high,” “low,” and no HHV‐6 detected), subgroup‐level testing was performed, as opposed to group‐level testing strictly by disease. In the AIC‐step optimization using PathSeq scores ∼ Cohort Classification coded by HHV‐6 status + Sex + Age + Source + Country, only cohort classification (phenotype) was a statistically significant covariate that remained in the model for PathSeq scores (Figure S1). Given that no other covariates were significant, PathSeq scores were inspected and used directly in subsequent analyses, including Fisher's exact test. Importantly, there was good agreement in PathSeq scores between blood and cerebellum for cohorts with WGSs derived from both sample types, with no significant difference in PathSeq scores for all viruses analyzed being replicated across both WGS sample sources in cohorts with adequate proportions derived from both blood and cerebellum (control, AD, FTD/ALS).

**FIGURE 1:**
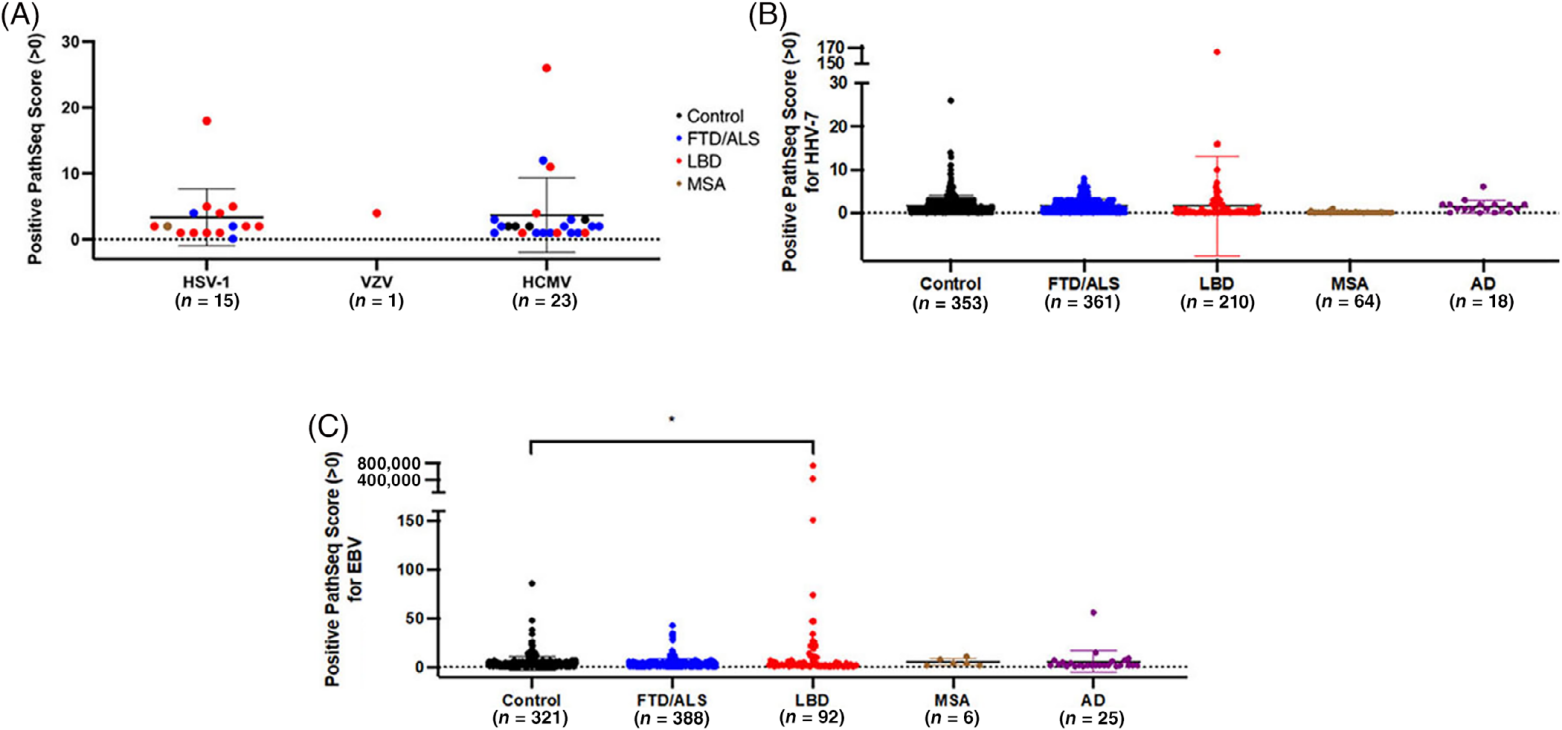
Integrated herpesviruses were detected in control and dementia cohorts. (A) Few whole‐genome sequences (WGSs) were positive for HSV‐1, VZV, and HCMV integration, with low PathSeq scores between 1 and 10. (B) Positive PathSeq scores for HHV‐7 were present in all cohorts, with most positive PathSeq scores less than 10. (C) Positive PathSeq scores for EBV were found in all cohorts. While most of the positive PathSeq scores were less than 10, there were also higher PathSeq scores between 10 and 151 in all cohorts. There were two PathSeq scores in the LBD cohort over 400,000. The mean positive PathSeq for EBV was significantly higher in the LBD cohort than in the control (*p = 0.01) (unpaired *t*‐test). Data are represented as the mean positive PathSeq scores ± SD. HCMV, human cytomegalovirus; HSV‐1, herpes simplex virus type 1; VZV, varicella zoster virus.

The neurologically relevant herpesviruses of interest in this study were human alphaherpesvirus 1 (herpes simplex virus type 1, HSV‐1), human alphaherpesvirus 2 (herpes simplex virus type 2, HSV‐2), human alphaherpesvirus 3 (varicella‐zoster virus, VZV), human betaherpesvirus 5 (cytomegalovirus, HCMV), human betaherpesvirus 6A (HHV‐6A), human betaherpesvirus 6B (HHV‐6B), human betaherpesvirus 7 (HHV‐7), and human gammaherpesvirus 4 (EBV). HSV‐2 was not detected by PathSeq in any WGS. Across all cohorts, few WGSs were positive for HSV‐1 (*n* = 15), VZV (*n* = 1), and HCMV (*n* = 23) (Figure 1A). PathSeq scores for these viruses were low, with the highest score being 26 for HCMV from a LBD patient. Most of the positive PathSeq scores for these viruses were between 1 and 10. There was no significant difference in the mean positive PathSeq scores between control and disease cohorts for HSV‐1 and HCMV (*p* > 0.05, unpaired *t* tests).

Positive PathSeq scores for HHV‐7 were found in all cohorts (control *n* = 353, FTD/ALS *n* = 361, LBD *n* = 210, MSA *n* = 64, AD *n* = 18) (Figure 1B). Most of the positive PathSeq scores for HHV‐7 were less than 10. The highest PathSeq score was 165 and was present in the LBD cohort. There was no significant difference in the mean positive PathSeq scores between control and dementia cohorts for HHV‐7 (*p* > 0.05, unpaired *t* tests).

With regard to EBV, positive PathSeq scores were also found in all cohorts (control *n* = 321, FTD/ALS *n* = 388, LBD *n* = 92, MSA *n* = 6, AD *n* = 25) (Figure 1C). While most of the positive PathSeq scores were less than 10, there were also higher PathSeq scores between 10 and 151 in all cohorts. Notably, there were two LBD samples with PathSeq scores over 400,000. The mean positive PathSeq score for EBV was significantly higher for the LBD cohort than the control cohort (*p* = 0.01, unpaired *t* test, *t* = 2.572, degrees of freedom (df) = 411). With the two outliers with PathSeq scores greater than 400,000 removed from the analysis for the LBD cohort, this significance remained (*p* = 0.0019, unpaired *t* test, *t* = 3.129, df = 409), suggesting that, on average, LBD WGS positive for EBV had higher PathSeq scores and therefore more microbe‐derived reads than WGSs in the control cohort with positive PathSeq scores for EBV.

**FIGURE 2:**
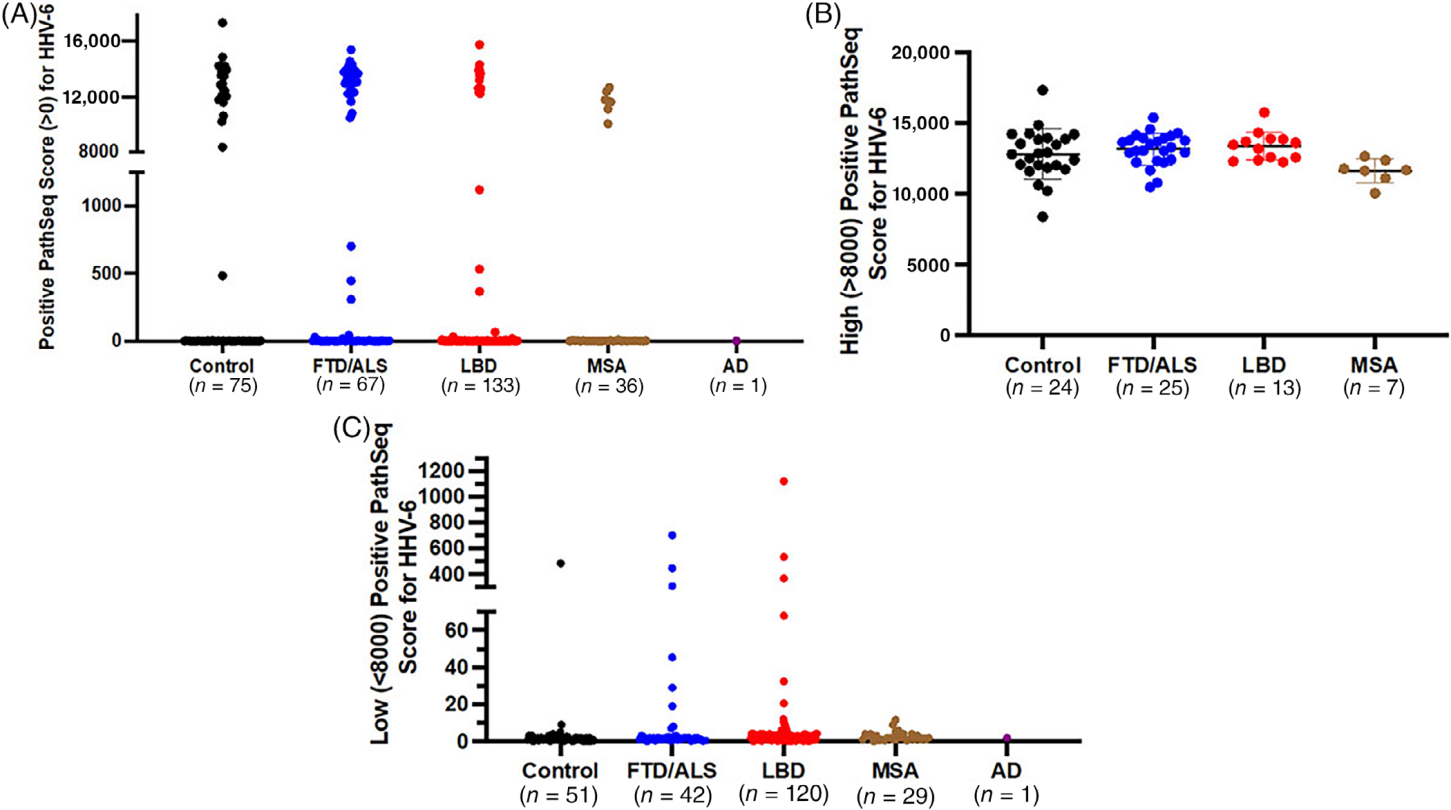
High and low PathSeq scores for HHV‐6 were detected in control and dementia cohorts. (A) Positive PathSeq scores for HHV‐6 were present in all cohorts. There was a cluster of higher PathSeq scores greater than 8000 in all cohorts except AD. (B) High PathSeq scores (>8000) for HHV‐6 were present in control, FTD/ALS, LBD, and MSA cohorts. Data are represented as the mean positive PathSeq score for each cohort ± SD. (C) Low positive PathSeq scores (< 8000) for HHV‐6 were present in all cohorts. AD, Alzheimer's disease; ALS, amyotrophic lateral sclerosis; FTD, frontotemporal dementia; HHV‐6, human herpesvirus‐6; LBD, Lewy body dementia; MSA, multiple system atrophy; SD, standard deviation.

Positive PathSeq scores for HHV‐6 were present in all cohorts (control *n* = 75, FTD/ALS *n* = 67, LBD *n* = 133, MSA *n* = 36, AD *n* = 1) (Figure 2A). Many WGSs had positive PathSeq scores for both HHV‐6A and HHV‐6B, likely due to the 90% nucleotide sequence identity between the two variants.^24^ Therefore, due to this inability to specifically distinguish each viral variant in all WGSs, prevalence analyses for HHV‐6 included WGSs with positive PathSeq scores for HHV‐6A or HHV‐6B. In some samples, the WGSs had positive PathSeq scores for HHV‐6, but one variant (either HHV‐6A or HHV‐6B) was significantly higher than the other. In those cases, the highest PathSeq score was used in analyses. While most of the positive PathSeq scores for HHV‐6 were low, there was also a cluster of high PathSeq scores greater than 8000 in all cohorts except AD (Figure 2B). Clusters of such high PathSeq scores were unique to HHV‐6 and were considered suggestive of iciHHV‐6 due to the large number of HHV‐6 reads in the WGSs. Most positive PathSeq scores for HHV‐6 were low and less than 8000. For subsequent analyses, WGS with positive PathSeq scores for HHV‐6 were arbitrarily classified as “high” (>8000) or “low” scores (<8000), based on the initial observed stratification of HHV‐6 PathSeq scores and selection of a value that distinguished the two classes. There was no significant difference in the mean positive PathSeq scores less than 8000 for HHV‐6 between cohorts (Figure 2C). There were no significant differences in the mean high or low HHV‐6 PathSeq scores between the control and disease cohorts (*p* > 0.05, unpaired *t* tests).

### 3.2 HHV‐6 PathSeq scores greater than 8000 were predictive of iciHHV‐6

**FIGURE 3:**
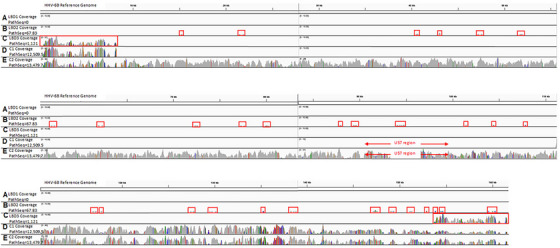
IGV highlighted the integration of segments of the HHV‐6 genome in samples with low PathSeq scores for HHV‐6. A subset of WGSs were analyzed using IGV, displaying the WGS coverage to the HHV‐6B reference genome. (A) LBD 1 WGSs had a PathSeq of 0 for HHV‐6B and no coverage of the HHV‐6B reference genome. (B) In LBD 2, the low PathSeq score of 67.83 was consistent with the integration of variably sized and randomly dispersed segments scattered along the HHV‐6B viral genome (red boxes). (C) A WGS with a low PathSeq score of 1121, LBD 3, was consistent with coverage and integration of the direct repeat (DR) segments of the HHV‐6B genome (red boxes). (D) Control 1 (C1), a WGS with a high PathSeq for HHV‐6B suggestive of iciHHV‐6B, did not have HHV‐6B detected by ddPCR due to the lack of integration of the amplified U57 region of the viral genome. (E) In Control 2 (C2), IGV confirmed full coverage of viral genome, including the ddPCR‐amplified U57 region, in a WGS with a high PathSeq score and ddPCR results of approximately one copy of HHV‐6B/cell. ddPCR, droplet digital polymerase chain reaction; HHV‐6, human herpesvirus‐6; IGV, Integrative Genomics Viewer; LBD, Lewy body dementia; WGS, whole‐genome sequence.

High PathSeq scores greater than 8000 for HHV‐6A or HHV‐6B were considered suggestive of iciHHV‐6 due to the large number of viral read counts in the WGSs. Of the 69 total WGSs with high PathSeq scores for HHV‐6 from all cohorts (69/7547 = 0.91%) (control *n* = 24, FTD/ALS *n* = 25, LBD *n* = 13, MSA *n* = 7), DNA was available from 29 samples for analysis with a well‐described ddPCR assay to quantify copies of HHV‐6 (variant A or B) for confirmation of iciHHV‐6 (Table 2).^34^, ^40^ Of these 29 samples, 27 (93%) were strongly positive for HHV‐6 at levels between 0.87 and 1.14 copies of HHV‐6 per cell (mean = 0.97 copies/cell) (Table S1). Such high ddPCR copies have been reported to indicate iciHHV‐6.^34^, ^40^, ^52^ Nine samples were shown to be iciHHV‐6A, and 18 were iciHHV‐6B, concordant with the higher HHV‐6 PathSeq score for the respective variant (Table S1). A subset of samples with high HHV‐6 PathSeq scores and ddPCR results of approximately one copy of HHV‐6 per cell, suggestive of iciHHV‐6, were also analyzed with the IGV to visualize the portions of the HHV‐6 genome that were present in the WGSs.^53^ The IGV displays the coverage track and the number of reads at each locus as a bar graph. These bars are gray if they match the reference genome; colors are used to highlight mismatches to the reference genome. A representative sample with a high PathSeq score for HHV‐6B and high ddPCR results of approximately 1 HHV‐6B copy per cell is shown in Figure 3E, which confirms the integration of the HHV‐6B full‐length genome in this individual.

**TABLE 2:** Summary of PathSeq scores and ddPCR results for the subset of tested samples.

Cohort	PathSeq score classification	Virus species	Number of samples	ddPCR results
Control *n* = 17	High	HHV‐6A	7	100% (7/7) consistent with iciHHV‐6A
		HHV‐6B	10	90% (9/10) consistent with iciHHV‐6B
				10% (1/10) not detected
Lewy body dementia (LBD) *n* = 13	Low	HHV‐6B	2	100% (2/2) not detected
	High	HHV‐6A	2	100% (2/2) consistent with iciHHV‐6A
		HHV‐6B	9	100% (9/9) consistent with iciHHV‐6B
Multiple system atrophy (MSA) *n* = 1	High	HHV‐6B	1	100% (1/1) notdetected

Abbreviations: HHV, human herpesvirus; iciHHV, inherited chromosomally integrated HHV‐6

Two samples with high PathSeq scores greater than 8000 did not have HHV‐6 detected by ddPCR (Table 2). This was explained by WGS analysis with IGV that demonstrated large portions of HHV‐6 present but absence of the viral U57 region (Figure 3D). As this is the portion of the viral genome amplified by the ddPCR primers and tagged by the fluorescent probes, it clarifies the lack of PCR reactivity in these samples. Two samples with low PathSeq scores of less than 8000 for HHV‐6 were also analyzed via ddPCR, but the virus was not detected in these samples with ddPCR.

**FIGURE 4:**
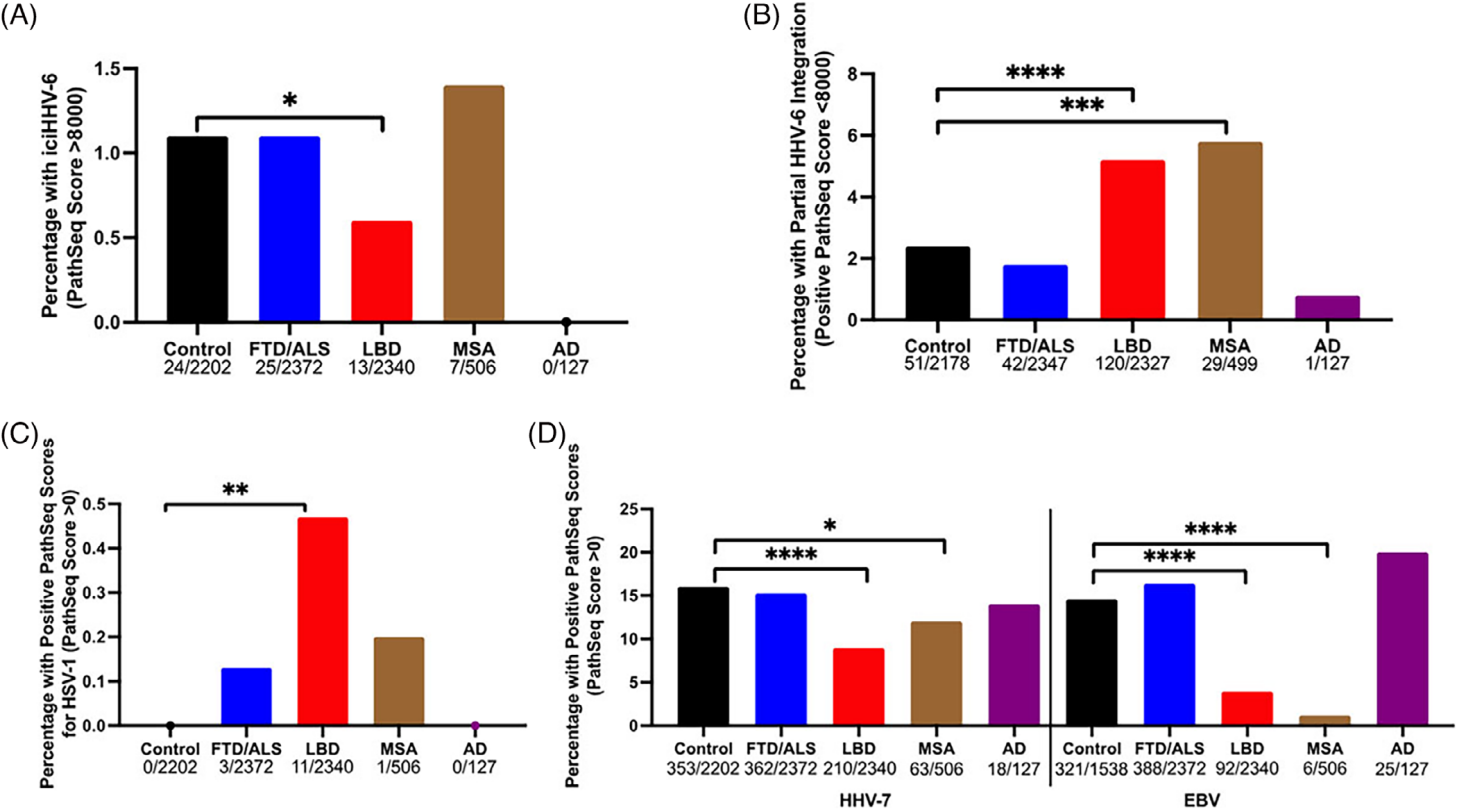
Partial HHV‐6 integration was more prevalent in synucleinopathy cohorts. (A) A lower prevalence of iciHHV‐6 was associated with the LBD cohort compared to the control cohort (*p = 0.048, Fisher's exact test). (B) A higher prevalence of partial HHV‐6 integration was associated with LBD and MSA cohorts when compared to the control cohort (****p < 0.0001, ***p = 0.0002; Fisher's exact test). (C) A higher prevalence of HSV‐1 was associated with the LBD cohort when compared to the control group (**p = 0.001; Fisher's exact test). (D) A lower prevalence of HHV‐7 integration was associated with the LBD cohort when compared to controls (****p < 0.0001, Fisher's exact test). The MSA cohort was also associated with a significantly lower HHV‐7 prevalence than controls (*p = 0.047, Fisher's exact test). With regard to EBV, the MSA and LBD cohorts were associated with a significantly lower prevalence than the controls (****p < 0.0001, Fisher's exact test). EBV, Epstein‐Barr virus; HHV‐6, human herpesvirus‐6; iciHHV inherited chromosomally integrated human herpesvirus; LBD, Lewy body dementia; MSA, multiple system atrophy;

The prevalence of iciHHV‐6 based on PathSeq scores of HHV‐6 greater than 8000 ranged from 0% to 1.4% in each cohort (Figure 4A). When comparing each cohort to the controls, a lower prevalence of iciHHV‐6 was present in the LBD group compared to the control cohort (*p* = 0.048, Fisher's exact test) (Figure 4A). While iciHHV‐6 is usually expected at a prevalence of about 1%, it has been documented to be as low as 0.2% in some populations.^26^ This may explain the lower prevalence of iciHHV‐6 in the LBD cohort and the lack of iciHHV‐6 in the comparatively smaller AD cohort.

### 3.3 Low PathSeq scores for HHV‐6 were consistent with the integration of small segments of viral genome, which was significantly more prevalent in LBD and MSA cohorts

Based on IGV analysis of a subset of WGSs and displayed coverage of the WGSs to the reference genomes, low positive PathSeq scores (less than 8000) for HHV‐6 were consistent with the suspected integration of variably sized segments of the viral genome or partial HHV‐6 integration. The majority of the 7547 (7235/7547; 95.9%) WGS samples had HHV‐6 PathSeq scores of 0 (Data S1). A representative sample is shown in Figure 3A, with no HHV‐6 viral genome coverage by IGV analysis. However, the analysis of a subset of samples with low PathSeq scores for HHV‐6 demonstrated possible partial integration with segments that varied from small randomly scattered portions of the viral genome (Figure 3B, red boxes) to more defined regions of HHV‐6, such as the DR regions present at each end of the HHV‐6 viral sequence (Figure 3C). The magnitude of the low PathSeq scores was roughly correlated with the amount of the reference viral genome present in the WGS.

The prevalence of partial HHV‐6 genome integration based on positive PathSeq scores for HHV‐6 less than 8000 ranged from 0.8% to 5.8% in each cohort (Figure 4B). Of interest is the observation that a statistically higher prevalence of partial HHV‐6 integration was associated with the LBD (*p* < 0.0001) and MSA (*p* = 0.0002) cohorts when compared to the control group (Fisher's exact test). No such increase was observed in the FTD/ALS and AD cohorts.

### 3.4 Higher prevalence in both synucleinopathies was specific to HHV‐6 partial integration

In addition to HHV‐6, other herpesviruses, including HSV‐1, VZV, HCMV, EBV, and HHV‐7, were also investigated for the presence of possible viral genome integration via the PathSeq pipeline in the 7547 WGSs from the control, FTD/ALS, LBD, MSA, and AD cohorts. As shown in Figure 4C, the prevalence of HSV‐1 integration in cohorts was very low compared to the partial integration of HHV‐6 (Figure 4B), ranging from 0% to 0.47%. Although there was a higher percentage of HSV‐1 integration in the LBD cohort, this was still low (0.47%) and was only significant since the control cohort had no HSV‐1‐related reads. Likewise, VZV‐related integrations were not present in the control cohort, and only one positive PathSeq score for VZV was found in the LBD cohort (0.04% prevalence). Similar to HSV‐1, the prevalence of HCMV in cohorts was also very low and ranged from 0% to 0.55%, with no significant differences in prevalence between the control and disease groups (Figure S2).

The prevalence of HHV‐7 and EBV was generally higher in the control cohort (Figure 4D) than partial HHV‐6 integration (Figure 4B) and ranged from 1% to 20% in all groups. However, no dementia‐related group had a significantly higher prevalence than controls. Indeed, there was a lower prevalence of HHV‐7 and EBV integration in the LBD (****p < 0.0001, Fisher's exact test) and MSA (*p = 0.047. *****p < 0.0001; Fisher's exact test) cohorts when compared to the control group, although the biological significance of this observation is unclear. Collectively, these observations suggest that the increase in HHV‐6 partial integrations in WGS of the LBD and MSA cohorts (Figure 4B) was specific compared to other herpesviruses.

## 4 DISCUSSION

The association of herpesviruses in neurodegenerative diseases, including AD and related dementias, has recently garnered renewed interest.^1^, ^2^, ^37^, ^54^ Whether these infectious agents play a role in disease pathogenesis or progression remains highly debated, with studies both supporting and refuting an association between viruses like HSV‐1 and HHV‐6 and AD.^14^, ^34^, ^35^, ^37^ In addition to their infectious properties, herpesviruses, specifically HHV‐6, have a unique ability to integrate into the host's genome.^8^, ^20^, ^21^, ^22^ In general, viral integration into host chromosomes can have a multitude of effects, including gene disruption and epigenetic changes.^8^ A recent study highlights that variation in the genetic sequences of viruses, including HHV‐6, may result in human genome structural variation.^55^

This study aimed to characterize herpesvirus genome integration in neurodegenerative disease and dementia, with a focus on Alzheimer's disease and related dementias like LBD and FTD/ALS. The comparatively smaller size of the AD cohort in this study limits the interpretation of the results in relation to AD, with an aim of expanding the analysis to larger AD cohorts in future studies. Using the PathSeq tool in the Broad Institute GATK, WGSs from patients in the control, FTD/ALS, LBD, MSA, and AD cohorts were screened for possible herpesvirus genome integration.^38^, ^39^ The resulting PathSeq scores are indirect measures of microbe prevalence and abundance, with “high” positive scores in the thousands being stronger evidence that the microbe is present based on the number of reads that align to the reference sequence.^14^, ^34^, ^38^, ^56^ In this study, positive PathSeq scores for HHV‐6 were unique in that they stratified into two clusters: high PathSeq scores greater than 8000 and low PathSeq scores greater than 0 but less than 8000.

Based on the number of corresponding viral reads in the WGS, high PathSeq scores for HHV‐6 greater than 8000 were suggestive of iciHHV‐6, the integration of the full‐length viral genome into the chromosomes of every nucleated cell in the body. IciHHV‐6 was confirmed via ddPCR in a subset of these samples with available DNA, with approximately one copy of virus per cell. The two samples with no detected HHV‐6 via ddPCR but high PathSeq scores had large portions of the viral genome integrated but were lacking the genomic sequence associated with the U57 major capsid protein, which was targeted by the ddPCR assay. While the LBD cohort was associated with a lower prevalence of iciHHV‐6 compared to the control cohort, prevalence in all cohorts was within previously reported ranges from varying geographical and clinical patient populations.^57^ The biological significance of the lower prevalence of iciHHV‐6 in the LBD cohort is unclear and may reflect a sampling effect. While at least one individual (about 1%) with a high PathSeq score for HHV‐6 and iciHHV‐6 may have been expected in the AD cohort, none were detected, likely due to the comparatively small sample size and the fact that documented prevalence of iciHHV‐6 can be as low as 0.2% based on the patient population.^26^ Whether the chromosomal integration of a viral genome may play a role in disease manifestation in these individuals with iciHHV‐6 through integration into telomeres on specific chromosomes or even outside the telomeric region is unknown.^32^, ^57^, ^58^, ^59^, ^60^ Additionally, reactivation of the virus in patients with iciHHV‐6 cannot be ruled out as a contributor to disease manifestation and progression, with previous evidence of HHV‐6 RNA expression in the brains of some iciHHV‐6 individuals.^20^, ^61^, ^62^, ^63^, ^64^ While the prevalence of iciHHV‐6 did not vary significantly in most cohorts, the role iciHHV‐6 may play in neurodegenerative disease and dementia in the individual patient is unclear.

Of interest in this study is the occurrence of low PathSeq scores for HHV‐6 that are consistent with partial integration of HHV‐6, where small portions of the viral genome may be present within the host, particularly in the LBD and MSA cohorts. Whether these viral genomic segments are remnants from iciHHV‐6 viral genome excision or viral reactivation is unknown.^32^, ^55^ Similar to our study, the retention of solely the DR regions of HHV‐6 was previously reported.^32^ HHV‐6 miRNAs can be expressed from viral segments, including the DRs, suggesting that expression of integrated segments of HHV‐6 may contribute to disease progression or changes to the host cells.^32^, ^65^ Additionally, whether these segments affect telomere length and stability, which has been documented in iciHHV‐6 and has been associated with HHV‐6 infection, is unknown.^66^, ^67^ Future studies are needed to experimentally confirm and characterize the sites of integration of these suspected viral genome segments, how the partial integration of HHV‐6 occurs, and the role partial HHV‐6 integration may be playing in dementia.

LBD and MSA, the cohorts associated with a significantly higher prevalence of low PathSeq scores and partial integration of HHV‐6, have similar clinical and pathological characteristics. Both LBD and MSA can have overlapping clinical features, including cognitive, movement, and autonomic dysfunction.^68^ They are both synucleinopathies, with hallmark α‐synuclein (α‐syn) aggregates found mainly in neurons in LBD and in glial cells in MSA.^69^ While the pathogenesis and factors involved in LBD and MSA manifestation and progression remain mostly unknown, recent studies highlight overlapping α‐syn co‐pathology in some cases of AD.^51^, ^70^, ^71^ In the few studies investigating the role of viruses on α‐syn aggregation, α‐syn has been shown to have a protective role against viral infection, similar to the hypothesized role of Aβ as an anti‐microbial peptide.^72^, ^73^, ^74^ In addition to infectious viruses potentially contributing to these diseases, this study highlights the possibility that HHV‐6 genome integration may play a role in a subset of MSA and LBD patients. A higher prevalence in the LBD and MSA cohorts was not noted in the other herpesviruses of interest in this study, suggesting a level of specificity for HHV‐6 partial integration in the LBD and MSA cohorts. Based on the results of this study, herpesvirus genome integration may play a unique role in LBD and MSA pathogenesis, and additional investigation into the role that herpesviruses play in these diseases is likely warranted.^75^

This study leveraged the publicly available PathSeq tool to characterize herpesvirus genome integration in dementia and control cohorts, finding a significantly higher prevalence in the partial integration of HHV‐6 in the LBD and MSA cohorts. This suggests a possible role for viral genome integration in a subset of these patients. This observation can be strengthened by replicating the PathSeq analysis in different cohorts of these same diseases, including a larger number of AD WGSs, and by extending the analysis to other neurodegenerative diseases. Additionally, future studies should focus on the mechanisms by which this herpesvirus genome integration could contribute to the development and pathogenesis of neurodegenerative disease and dementia.

## CONFLICT OF INTEREST STATEMENT

Sonja W. Scholz serves on the scientific advisory board of the Lewy Body Dementia Association, the Multiple System Atrophy Coalition, and G‐Can. Sonja W. Scholz is an editorial board member for the *Journal of Parkinson's Disease* and *JAMA Neurology*. Sonja W. Scholz and Bryan J. Traynor receive research support from Cerevel Therapeutics. Bryan J. Traynor is an editorial and advisory board member for *Brain, eClinicalMedicine, Journal of Neurology, Neurosurgery and Psychiatry*, and *Neurobiology of Aging*. Bryan J. Traynor holds US, EU, and Canadian patents on the clinical testing and therapeutic intervention for the hexanucleotide repeat expansion of C9orf72. All other authors declare no competing interests. Author disclosures are available in the Supporting Information.

## CONSENT STATEMENT

Review boards of participating institutions approved the study (NIH, NIA, study number: 03‐AG‐N329). Informed consent was obtained from subjects or their surrogate decision‐makers, according to the Declaration of Helsinki.

## Supporting information



Supporting Information

Supporting Information

Supporting Information

Supporting Information

## Data Availability

All data supporting the findings of this study are provided within the article and the supplementary files. A file including the complete PathSeq results generated during this study is available for download at https://data.ninds.nih.gov/. The genome data are available on dbGaP (https://www.ncbi.nlm.nih.gov/gap/), accession number phs001963.v3.p1. All additional information and material requests should be addressed to the corresponding author.

## 1 COLLABORATORS

### 1.1 International LBD Genomics Consortium

(Principal investigators for individual study sites are separated by country)

**Canada**: *Sandra E. Black* (Institute of Medical Science, Faculty of Medicine, University of Toronto, Toronto, ON, Canada; Division of Neurology, Department of Medicine, University of Toronto, Toronto, ON, Canada; Heart and Stroke Foundation Canadian Partnership for Stroke Recovery, Sunnybrook Health Sciences Centre, University of Toronto, Toronto, ON, Canada; Hurvitz Brain Sciences Research Program, Sunnybrook Research Institute, University of Toronto, Toronto, ON, Canada; LC Campbell Cognitive Neurology Research Unit, Sunnybrook Research Institute, University of Toronto, Toronto, ON, Canada), *Ziv Gan‐ Or* (Montreal Neurological Institute and Hospital, Department of Neurology & Neurosurgery, McGill University, Montreal, Canada), *Julia Keith* (Department of Anatomical Pathology, Sunnybrook Health Sciences Centre, University of Toronto, Toronto, ON, Canada), *Mario Masellis* (Cognitive & Movement Disorders Clinic, Sunnybrook Health Sciences Centre, University of Toronto, Toronto, ON, Canada; Division of Neurology, Department of Medicine, University of Toronto, Toronto, ON, Canada; Hurvitz Brain Sciences Research Program, Sunnybrook Research Institute, University of Toronto, Toronto, ON, Canada; LC Campbell Cognitive Neurology Research Unit, Sunnybrook Research Institute, University of Toronto, Toronto, ON, Canada), *Ekaterina Rogaeva* (Tanz Centre for Research in Neurodegenerative Diseases, University of Toronto, Toronto, ON, Canada).

**France**: *Alexis Brice* (Sorbonne University, Institute Du Cerveau – Paris Brain Institute, Paris, France), *Suzanne Lesage* (Sorbonne Universites, Institute Du Cerveau – Paris Brain Institute, Paris, France).

**Greece**: *Georgia Xiromerisiou* (Department of Neurology, University of Thessalia, University Hospital of Larissa, Larissa, Greece).

**Italy**: *Andrea Calvo* (“Rita Levi Montalcini” Department of Neuroscience, University of Turin, Turin, Italy), *Antonio Canosa* (“Rita Levi Montalcini” Department of Neuroscience, University of Turin, Turin, Italy), *Adriano Chio* (“Rita Levi Montalcini” Department of Neuroscience, University of Turin, Turin, Italy; Institute of Cognitive Sciences and Technologies, C.N.R., Rome, Italy; Azienda Ospedaliero Universitaria Città della Salute e della Scienza, Turin, Italy), *Giancarlo Logroscino* (Center for Neurodegenerative Diseases and the Aging Brain, University of Bari Aldo Moro At Pia Fondazione Panico Hospital‐ Tricase (LE), Bari, Italy), *Gabriele Mora* (ALS Center, Istituti Clinici Scientifici Maugeri, IRCCS Milano, Milan, Italy).

**Luxembourg**: *Reijko Krüger* (Luxembourg Center for Systems Biomedicine, University of Luxembourg, Esch‐sur‐Alzette, Luxembourg; Transversal Translational Medicine, Luxembourg Institute of Health, Strassen Luxembourg; Parkinson Research Clinic, Centre Hospitalier de Luxembourg, Luxembourg), *Patrick May* (Luxembourg Center for Systems Biomedicine, University of Luxembourg, Esch‐sur‐Alzette, Luxembourg).

**Spain**: *Daniel Alcolea* (Sant Pau Biomedical Institute, Hospital de la Santa Creu i Sant Pau, Universitat Autònoma de Barcelona, Barcelona, Spain; Network Center for Biomedical Research in Neurodegenerative Diseases (CIBERNED), Madrid, Spain), *Jordi Clarimon* (Sant Pau Biomedical Research Institute, Hospital de la Santa Creu i Sant Pau, Universitat Autònoma de Barcelona, Barcelona, Spain; Network Center for Biomedical Research in Neurodegenerative Diseases (CIBERNED), Madrid, Spain), *Juan Fortea* (Sant Pau Biomedical Research Institute, Hospital de la Santa Creu i Sant Pau, Universitat Autònoma de Barcelona, Barcelona, Spain; Network Center for Biomedical Research in Neurodegenerative Diseases [CIBERNED], Madrid, Spain), *Isabel Gonzalez‐Aramburu* (Institute for Research Marqués (IDIVAL), University of Cantabria and Department of Neurology, Marqués de Valdecilla Hospital, Santander, Spain; Centro de Investigación Biomédica en Red sobre Enfermedades Neurodegenerativas (CIBERNED), Madrid, Spain), *Jon Infante* (Institute for Research Marqués (IDIVAL), University of Cantabria and Department of Neurology, Marqués de Valdecilla Hospital, Santander, Spain; Centro de Investigación Biomédica en Red sobre Enfermedades Neurodegenerativas (CIBERNED), Madrid, Spain), *Carmen Lage* (Institute for Research Marqués (IDIVAL), University of Cantabria and Department of Neurology, Marqués de Valdecilla Hospital, Santander, Spain; Centro de Investigación Biomédica en Red sobre Enfermedades Neurodegenerativas (CIBERNED), Madrid, Spain), *Alberto Lleó* (Sant Pau Biomedical Research Institute, Hospital de la Santa Creu I Sant Pau, Universitat Autònoma de Barcelona, Barcelona, Spain; The Network Center for Biomedical Research in Neurodegenerative Diseases (CIBERNED), Madrid, Spain), *Pau Pastor* (Memory and Movement Disorders Units, Department of Neurology, University Hospital Mutua de Terrassa, Barcelona, Spain), *Pascual Sanchez‐Juan* (Institute for Research Marqués (IDIVAL), University of Cantabria and Department of Neurology, Marqués de Valdecilla Hospital, Santander, Spain; Centro de Investigación Biomédica en Red sobre Enfermedades Neurodegenerativas (CIBERNED), Madrid, Spain).

**Republic of Ireland**: *Francesca Brett* (Dublin Brain Bank, Neuropathology Department, Beaumont Hospital, Dublin, Ireland).

**United Kingdom**: *Dag Aarsland* (Institute of Psychiatry, Psychology and Neuroscience (IoPPN), King's College London, London, UK), *Safa Al‐Sarraj* (Department of Clinical Neuropathology, King's College Hospital and London Neurodegenerative Diseases Brain Bank, Institute of Psychiatry, Psychology and Neuroscience (IoPPN), King's College London, London, UK), *Johannes Attems* (Translational and Clinical Research Institute, Campus for Ageing and Vitality, Newcastle University, Newcastle upon Tyne, UK), *Steve Gentleman* (Neuropathology Unit, Department of Brain Sciences, Imperial College London, London, UK), *John A. Hardy* (Department of Neurodegenerative Disease, UCL Queen Square Institute of Neurology, Queen Square, London, UK; UK Dementia Research Institute at University College London, UCL Institute of Neurology, University College London, London, UK; Reta Lila Weston Institute, UCL Queen Square Institute of Neurology, London, UK; UCL Movement Disorders Centre, University College London, London, UK), *Angela K. Hodges* (Institute of Psychiatry, Psychology and Neuroscience (IoPPN), King's College London, London, UK), *Seth Love* (Dementia Research Group, School of Clinical Sciences, University of Bristol, Southmead Hospital, Bristol, UK), *Ian G. McKeith* (Translational and Clinical Research Institute, Campus for Ageing and Vitality, Newcastle University, Newcastle upon Tyne, UK), *Christopher M. Morris* (Translational and Clinical Research Institute, Campus for Ageing and Vitality, Newcastle University, Newcastle upon Tyne, UK), *Huw R. Morris* (Department of Clinical and Movement Neuroscience, UCL Queen Square Institute of Neurology, University College London, London, UK), *Laura Palmer* (South West Dementia Brain Bank, University of Bristol, Southmead Hospital, Bristol, UK), *Stuart Pickering‐Brown* (Division of Neuroscience and Experimental Psychology, Faculty of Biology, Medicine and Health, University of Manchester, Manchester, UK), *Mina Ryten* (NIHR Great Ormond Street Hospital Biomedical Research Centre, University College London, London, UK; Genetics and Genomic Medicine, Great Ormond Street Institute of Child Health, University College London, London, UK), *Alan J. Thomas* (Biomedical Research Building, Campus for Aging and Vitality, Newcastle University, Newcastle upon Tyne, UK), *Claire Troakes* (Institute of Psychiatry, Psychology and Neuroscience (IoPPN), King's College London, London, UK).

**United States of America**: *Marilyn S. Albert* (Department of Neurology, Johns Hopkins University Medical Center, Baltimore, MD, USA), *Matthew J. Barrett* (Department of Neurology, University of Virginia School of Medicine, Charlottesville, VA, USA), *Thomas G. Beach* (Banner Sun Health Research Institute, Sun City, AZ, USA), *Lynn M. Bekris* (Genomic Medicine Institute, Cleveland Clinic, Cleveland, OH, USA), *David A. Bennett* (Rush Alzheimer's Disease Center, Chicago, IL, USA), *Bradley F. Boeve* (Department of Neurology, Mayo Clinic, Rochester, MN, USA), *Clifton L. Dalgard* (Department of Anatomy, Physiology and Genetics, Uniformed Services University of the Health Sciences, Bethesda, MD, USA), *Ted M. Dawson* (Department of Neurology, Johns Hopkins University Medical Center, Baltimore, MD, USA; Neuroregeneration and Stem Cell Programs, Institute of Cell Engineering, Johns Hopkins University School of Medicine, Baltimore, MD, USA; Department of Pharmacology and Molecular Science, Johns Hopkins University School of Medicine, Baltimore, MD, USA; Solomon H. Snyder Department of Neuroscience, Johns Hopkins University School of Medicine, Baltimore, MD, USA), *Dennis W. Dickson* (Department of Neuroscience, Mayo Clinic, Jacksonville, FL, USA), *Kelley Faber* (Indiana University School of Medicine, Indianapolis, IN, USA), *Tanis Ferman* (Department of Psychiatry and Psychology, Mayo Clinic, Jacksonville, FL, USA), *Luigi Ferrucci* (Longitudinal Studies Section, National Institute on Aging, Baltimore, MD, USA), *Margaret E. Flanagan* (Northwestern University Feinberg School of Medicine, Chicago, IL, USA), *Tatiana M. Foroud* (Indiana University School of Medicine, Indianapolis, IN, USA), *Bernardino Ghetti* (Department of Pathology and Laboratory Medicine, Indiana University School of Medicine, Indianapolis, IN, USA), *J. Raphael Gibbs* (Laboratory of Neurogenetics, National Institute on Aging, MD, USA), *Alison Goate* (Ronald M. Loeb Center for Alzheimer's Disease, Nash Family Department of Neuroscience, Department of Genetics and Genomic Science, and Department of Pathology, Icahn School of Medicine at Mount Sinai, New York, NY, USA), *David S. Goldstein* (Clinical Neurocardiology Section, National Institute of Neurological Disorders and Stroke, Bethesda, MD, USA), *Neill R. Graff‐Radford* (Department of Neurology, Mayo Clinic Florida, Jacksonville, FL, USA), *Horacio Kaufmann* (Department of Neurology, New York University School of Medicine, NY, USA), *Walter A. Kukull* (National Alzheimer's Coordinating Center (NACC), University of Washington, Seattle, WA, USA), *James B. Leverenz* (Cleveland Lou Ruvo Center for Brain Health, Neurological Institute, Cleveland Clinic, Cleveland, OH, USA), *Qinwen Mao* (Northwestern University Feinberg School of Medicine, Chicago, IL, USA), *Eliezer Masliah* (Laboratory of Neurogenetics, National Institute on Aging, Bethesda, MD, USA), *Edwin Monuki* (University of California Irvine, Irvine, CA, USA), *Kathy L. Newell* (Department of Pathology and Laboratory Medicine, Indiana University School of Medicine, Indianapolis, IN, USA), *Jose‐Alberto Palma* (Department of Neurology, New York University School of Medicine, New York, NY, USA), *Matthew Perkins* (Department of Neurology, Michigan Medicine, University of Michigan, Ann Arbor, MI, USA), *Olga Pletnikova* (Department of Pathology (Neuropathology), Johns Hopkins University School of Medicine, Baltimore, MD, USA; Department of Pathology and Anatomical Sciences, Jacobs School of Medicine and Biomedical Sciences, University at Buffalo the State University of New York, USA), *Alan E. Renton* (Ronal M. Loeb Center for Alzheimer's Disease and Nash Family Department of Neuroscience, Icahn School of Medicine at Mount Sinai, New York, NY, USA), *Susan M. Resnick* (Laboratory of Behavioral Neuroscience, National Institute on Aging, Baltimore, MD, USA), *Liana S. Rosenthal* (Department of Neurology, Johns Hopkins University Medical Center, Baltimore, MD, USA), *Owen A. Ross* (Department of Neuroscience, Mayo Clinic, Jacksonville, FL, USA; Department of Clinical Genomics, Mayo Clinic, Jacksonville, FL, USA), *Clemens R. Scherzer* (Harvard Medical School and Brigham & Women's Hospital, Boston, MA, USA), *Geidy E. Serrano* (Banner Sun Health Research Institute, Sun City, AZ, USA), *Vikram G. Shakkottai* (Department of Neurology, University of Texas Southwestern Medical Center, Dallas, TX, USA), *Ellen Sidransky* (Medical Genetics Branch, National Human Genome Research Institute, Bethesda, MD, USA), *Toshiko Tanaka* (Longitudinal Studies Section, National Institute on Aging, Baltimore, MD, USA), *Eric Topol* (Scripps Research Translational Institute, Scripps Research, La Jolla, CA, USA), *Ali Torkamani* (Scripps Research Translational Institute, Scripps Research, La Jolla, CA, USA), *Juan C. Troncoso* (Department of Pathology (Neuropathology), Johns Hopkins University School of Medicine, Baltimore, MD, USA), *Randy Woltjer* (Department of Neurology, Oregon Health & Sciences University, Portland, OR, USA), *Zbigniew K. Wszolek* (Department of Neurology, Mayo Clinic Florida, Jacksonville, FL, USA), *Sonja W. Scholz* (Neurodegenerative Diseases Research Unit (National Institute of Neurological Disorder and Stroke), Bethesda, MD, USA; Department of Neurology, Johns Hopkins University Medical Center, Baltimore, MD, USA).

**Acknowledgements for consortium members**:

T.F. and K.F. report that samples provided by the National Centralized Repository for Alzheimer's Diseases and Related Dementias (NCRAD) were supported under a cooperative agreement grant (U24 AG021886). This study used tissue samples and data that were provided by the Johns Hopkins Morris K. Udall Center of Excellence for Parkinson's Disease Research (NIH P50 NS38377). Z.K.W. is partially supported by the NIH/NIA and NIH/NINDS (1U19AG063911, FAIN: U19AG063911), Mayo Clinic Center for Regenerative Medicine, gifts from the Donald G. and Jodi P. Heeringa Family, the Haworth Family Professorship in Neurodegenerative Diseases Fund, Albertson Parkinson's Research Foundation, PPND Family Foundation, and Margaret N. and John Wilchek Family. We are grateful to the Banner Sun Health Research Institute Brain and Body Donation Program of Sun City, Arizona, for the provision of human brain tissue and data. The Brain and Body Donation Program is supported by the National Institute of Neurological Disorders and Stroke (U24 NS072026 National Brain and Tissue Resource for Parkinson's Disease and Related Disorders), the National Institute on Aging (P30 AG19610 Arizona Alzheimer's Disease Core Center), the Arizona Department of Health Services (contract 211002, Arizona Alzheimer's Research Center), the Arizona Biomedical Research Commission (contracts 4001, 0011, 05‐901 and 1001 to the Arizona Parkinson's Disease Consortium) and the Michael J. Fox Foundation for Parkinson's Research. We are grateful to the Rush Alzheimer's Disease Center for providing brain tissue and DNA samples, which was supported by the grants P30 AG10161, R01 AG15819, R01 AG17917, U01AG46152, U01 AG61356.

**Conflict of interest statement for consortium members**:

Z.K.W. serves as PI or Co‐PI on Biohaven Pharmaceuticals, Inc. (BHV4157‐206), Vigil Neuroscience, Inc. (VGL101‐01.002, VGL101‐01.201, positron emission tomography tracer development protocol, Csf1r biomarker and repository project, and ultra‐high‐field magnetic resonance imaging in the diagnosis and management of CSF1R‐related adult‐onset leukoencephalopathy with axonal spheroids and pigmented glia), and ONO‐2808‐03 projects/grants. He serves as Co‐PI of the Mayo Clinic APDA Center for Advanced Research and as an external advisory board member for the Vigil Neuroscience, Inc., and as a consultant for Eli Lilly & Company and for NovoGlia, Inc.

## 2 The American Genome Center (TAGC)

Anthony R. Soltis^1^, Coralie Viollet^1^, Gauthaman Sukumar^1^, Camille Alba^1^, Nathaniel Lott^1^, Elisa McGrath Martinez^1^, Meila Tuck^1^, Jatinder Singh^1^, Dagmar Bacikova^1^, Xijun Zhang^1^ Daniel N. Hupalo^1^, Adelani Adeleye^1^, Matthew D. Wilkerson^1^, Harvey B. Pollard^1^, Clifton L. Dalgard^1,2^

^1^The American Genome Center, Collaborative Health Initiative Research Program, Uniformed Services University of the Health Sciences, Bethesda, Maryland, USA

^2^Department of Anatomy, Physiology & Genetics, Uniformed Services University of the Health Sciences, Bethesda, Maryland, USA
