# Supplementary material for: Herpesvirus genome integration in whole‐genome sequences of dementia and control cohorts
Source: Alzheimers Dement. 2026 Mar 19;22(3):e71047. doi: 10.1002/alz.71047 (PMC13093769; doi:10.1002/alz.71047)
Supplement: Supplementary file 2 — Supporting Information [file ALZ-22-e71047-s001.pdf]

Supplementary Information

Table S1: HHV-6 ddPCR results for the subset of samples tested.

Cohort	PathSeq Score Classification	Virus Species	PathSeq Score	ddPCR Results (copies/cell)
Control n = 17	High	HHV-6A (n = 7)	14,218.33	1.10
			11,749.67	0.90
			11,596.5	0.95
			12,393.67	0.93
			12,073	0.95
			11,840.33	0.96
			17,336	0.89
		HHV-6B (n = 10)	14,203.5	1.03
			12,509.5	Not detected
			13,479.67	1.09
			13,845.67	0.93
			13,934.5	0.95
			14,862.67	0.94
			13,540.5	1.04
			14,255.5	0.97
			12,856.83	0.95
			12,932	0.97
LBD n = 13	High	HHV-6A (n = 2)	12,233.5	0.98
			12,566.83	0.92
		HHV-6B (n = 9)	15,762.5	0.93
			12,399.17	1.04
			13,604.17	0.92
			13,853.67	1.14
			14,319.33	1.06
			13,469.17	0.87
			13,895.5	1.00
			13,195.83	0.95
			12,596.67	0.95
	Low	HHV-6B (n = 2)	1121	Not detected
			534	Not detected
MSA n = 1	High	HHV-6B (n = 1)	11,694.67	Not detected

a
```
Cerebellum and Blood vs Cx (Initial) for HHV6A:
                Sum Sq   Df  F value Pr(>F)
(Intercept)        438    1   0.0014 0.9696
CX          1290420230   13 328.3083 <2e-16 ***
GENDER           27635    1   0.0914 0.7624
AGE               7136    1   0.0236 0.8779
RACE            311055    7   0.1470 0.9943
SOURCE          238001    1   0.7872 0.3750
Residuals   2135175671 7062
---
Signif. codes:  0 '***' 0.001 '**' 0.01 '*' 0.05 '.' 0.1 ' ' 1
```

b
```
Cerebellum and Blood vs Cx (Final) for HHV6A:
                Sum Sq   Df F value Pr(>F)
(Intercept)          0    1    0.00      1
CX          1290781914   13  328.77 <2e-16 ***
Residuals   2135791071 7072
---
Signif. codes:  0 '***' 0.001 '**' 0.01 '*' 0.05 '.' 0.1 ' ' 1
```

c
```
Cerebellum and Blood vs Cx (Initial) for HHV6B:
                Sum Sq   Df   F value Pr(>F)
(Intercept)        516    1    0.0016 0.9684
CX          5857231321   13 1368.2309 <2e-16 ***
GENDER            2513    1    0.0076 0.9304
AGE              12170    1    0.0370 0.8476
RACE            367905    7    0.1596 0.9927
SOURCE          276126    1    0.8385 0.3598
Residuals   2325505367 7062
---
Signif. codes:  0 '***' 0.001 '**' 0.01 '*' 0.05 '.' 0.1 ' ' 1
```

d
```
Cerebellum and Blood vs Cx (Final) for HHV6B:
                Sum Sq   Df F value Pr(>F)
(Intercept)          0    1     0.0      1
CX          5859423461   13  1370.4 <2e-16 ***
Residuals   2326049718 7072
---
Signif. codes:  0 '***' 0.001 '**' 0.01 '*' 0.05 '.' 0.1 ' ' 1
```

e
```
Cerebellum and Blood vs Cx (Initial) for VZV:
             Sum Sq   Df F value    Pr(>F)
(Intercept)  0.0000    1  0.0000    0.9977
CX           0.1333   13  4.5682 7.347e-08 ***
GENDER       0.0042    1  1.8678    0.1718
AGE          0.0009    1  0.4017    0.5262
RACE         0.0026    7  0.1671    0.9916
SOURCE       0.0000    1  0.0072    0.9326
Residuals   15.8568 7062
---
Signif. codes:  0 '***' 0.001 '**' 0.01 '*' 0.05 '.' 0.1 ' ' 1
```

f
```
Cerebellum and Blood vs Cx (Final) for VZV:
             Sum Sq   Df F value    Pr(>F)
(Intercept)  0.0011    1  0.4912    0.4834
CX           0.1340   13  4.5959 6.342e-08 ***
GENDER       0.0046    1  2.0433    0.1529
Residuals   15.8610 7071
---
Signif. codes:  0 '***' 0.001 '**' 0.01 '*' 0.05 '.' 0.1 ' ' 1
```

Figure S1: Akaike information criterion (AIC)-step optimization using PathSeq scores ~ Cohort Classification coded by HHV-6 status (phenotype) + Sex (gender) + Age + Source (blood or cerebellum) + Country (race).

Following ANCOVA testing, three viruses (HHV-6A, HHV-6B, and VZV) had differential PathSeq scores across phenotype classes. For both HHV-6A and HHV-6B, in both the pre-AIC step optimized ANCOVA model (a & c) and the post-AIC step optimized ANCOVA model (b & d), phenotype was a statistically significant covariate and the only covariate remaining in the final model (b & d). In both the pre-AIC step optimized ANCOVA model for VZV (e) and the post-AIC step optimized ANCOVA model for VZV (f), phenotype was a statistically significant covariate. While gender (sex) remained in the final model for VZV (f), the effect was not statistically significant.

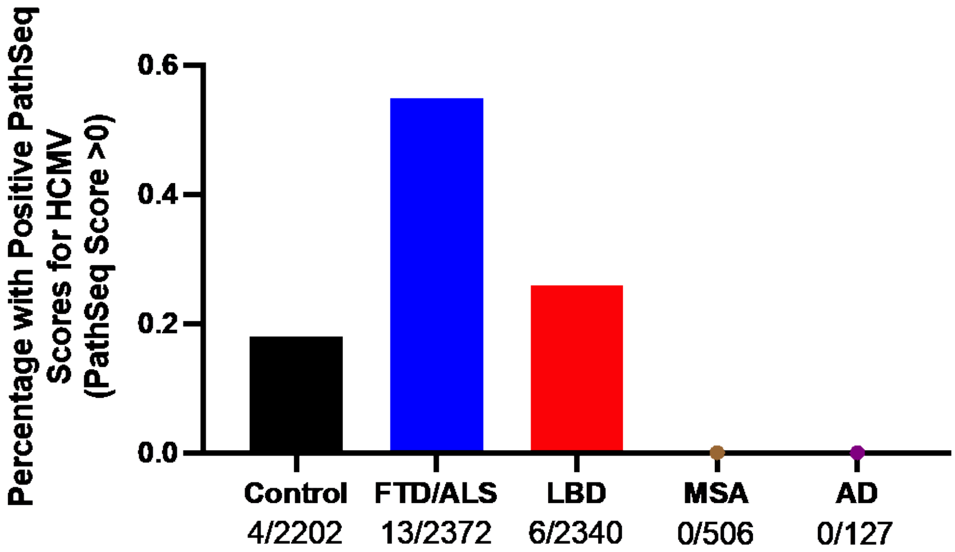

Figure S2: Prevalence of the Integration of HCMV based on PathSeq Score in Control and Dementia Cohorts

There were no differences in the prevalence of HCMV between control and dementia cohorts (p>0.05, Fisher's exact test).
